# Isolation and pathogenicity of a variant porcine epidemic diarrhea virus field strain with high adaptability to Vero cell

**DOI:** 10.3389/fvets.2025.1654230

**Published:** 2025-08-26

**Authors:** Tianjing Liao, Yi Zhang, Huanhuan Ma, Zhanchang Wang, Ao Zhou, Yu Zhang, Zhengfan Zhang, Shuangshuang Guo, Yongqing Hou, Zhonghua Li, Tao Wu

**Affiliations:** ^1^Hubei Key Laboratory of Animal Nutrition and Feed Science, Wuhan Polytechnic University, Wuhan, China; ^2^Forestry and Fruit Tree Research Institute, Wuhan Academy of Agricultural Sciences, Wuhan, China

**Keywords:** porcine epidemic diarrhea virus, G2b subtype, isolation, adaptability, pathogenicity

## Abstract

Since 2010, new outbreaks of porcine epidemic diarrhea (PED) caused by porcine epidemic diarrhea virus (PEDV) variant strains have brought significant economic losses to world pig industry. In this study, we isolated a PEDV strain from a new PED outbreak farm in 2024. The strain was identified through RT-PCR, indirect immunofluorescence assay and purified through plaque assay. This virus showed high adaptability to Vero cell during the process of passage and named as HB-2024. Phylogenetic analysis of the S gene showed that the HB-2024 strain was clustered into G2b subgroup. Amino sequence analysis showed that the S protein of the HB-2024 strain had a unique character beside the N terminal of the fusion peptide, which might lead to its high adaptability to Vero cell. We also performed a piglet infection experiment to test its pathogenicity. All piglets infected with this virus showed obvious diarrhea and their small intestines showed serious pathological damage. These results suggest that the HB-2024 strain is a G2b subtype variant that adapts well to Vero cell and can be used to study the adaptive mechanisms of PEDV.

## 1 Introduction

Porcine epidemic diarrhea (PED) is an intestinal disease characterized by vomiting, watery diarrhea, and dehydration, which is caused by the porcine epidemic diarrhea virus (PEDV). Due to its strong transmission, high morbidity and high mortality in piglets, this disease is currently a significant threat to the global pig industry (1, 2). PEDV is an enveloped virus that belongs to the family Coronaviridae and is a member of the genus Alphacoronavirus (3). The genome of PEDV is a single-stranded positive-sense RNA, with a length of about 28 Kb, comprising 5′ and 3′ untranslated regions as well as seven open reading frames (ORFs) (4). These ORFs encode four structural proteins: the spike (S) protein, the membrane (M) protein, the nucleocapsid (N) protein, and the envelope (E) protein; two replicase polyproteins: ORF1a and ORF1b; in addition to an accessory protein, ORF3.

The S protein, the largest structural protein of PEDV, is a type I transmembrane glycoprotein. This protein forms trimeric structures on the viral surface, playing a crucial role in PEDV entry into its host cells (5). The PEDV S protein is composed of 1,380 to 1,390 amino acids, with a molecular weight ranging from 180 to 200 kD (6). The S protein can be divided into two functionally distinct subunits: the N-terminal S1 subunit, which is involved in binding to cellular receptors, and the C-terminal S2 subunit, which mediates the membrane fusion between PEDV and its host cells (7). The S gene, which encodes the spike (S) protein, exhibits extremely high genetic variability and serves as a critical marker for genotyping PEDV as well as distinguishing virulent and attenuated strains (8).

PED was first reported in England in 1971 (9) and subsequently spread to various countries across Europe and Asia (10, 11). The first outbreak of PED in China was reported in 1973, but the isolation and identification of the PEDV was not achieved until 1984 (12). From 1973 to 2010, PED exhibited sporadic outbreaks in China, and most of these strains causing PED were later referred to as classical strains (13). However, after 2010, PED caused by PEDV variant strains broke out extensively in China, causing significant economic losses to the pig industry (13, 14). Phylogenetic tree analysis revealed that the variant strains had low homology with the classical strains and were located on distinctly different evolutionary branches. Therefore, from this time onwards, PEDV strains were divided into two genotypes, G1 and G2 (15). In 2013, a widespread outbreak of PED caused by G2 genotype occurred in the United States (16), causing serious losses to the pig industry. In 2014, Wang et al. (17) reported a novel USA PEDV strain (OH851), which was potentially formed through the recombination of G1 and G2 genotypes. Subsequently, researchers classified PEDV strains that exhibited sequence characteristics similar to OH851 into a new genotype, referred to as the S-INDEL genotype (18, 19). Additionally, the G1 genotype comprises two genetic subtypes, G1a and G1b (20), while the G2 genotype is divided into four genetic subtypes: G2a, G2b, G2c, and G2d (21). Currently, PED is prevalent globally, with the G2 genotype being the predominant circulating strains.

In this study, a PEDV G2b strain was isolated from intestinal samples of diarrheic piglets collected in Hubei Province, China, and designated as the HB-2024 strain. The complete genome of this virus was determined and the S gene sequence was analyzed. The results of cell experiments demonstrated that the HB-2024 strain exhibited high adaptability to Vero cells, while the results of animal experiment indicated that this strain could induce severe diarrhea in piglets.

## 2 Materials and methods

### 2.1 PEDV strains and cell line

Vero cells (renal cells from African green monkey) were purchased from Procell Life Science & Technology Co., Ltd (Wuhan, China) and cultivated in Dulbecco's modified Eagle's medium (DMEM; Cytiva, USA) supplemented with 10% fetal bovine serum (ExCell Bio, China) and 1% penicillin-streptomycin solution (Yeasen, China). PEDV strains YN (GenBank accession No. KT021228) and GD (GenBank accession No. KU985230), two previous isolated G2b strains, were propagated in Vero cells using DMEM containing 8 μg/ml trypsin and 1% penicillin-streptomycin solution.

### 2.2 Sample collection

Small intestine samples of two piglets suffering from severe diarrhea were gathered on a farm in Hubei province, China. An appropriate amount of tissue sample was taken and homogenized with DMEM at a ratio of 1:5 using a homogenizer. Following the process of freezing and thawing the tissue homogenate three times, the sample was subjected to centrifugation at a speed of 12,000 rpm for a duration of 10 min. After centrifugation, the supernatant was sterilized by filtration using a 0.22 μm filter. The filtered supernatant was then stored at a temperature of −80°C to preserve its integrity for future analyses.

### 2.3 RT-PCR

Viral RNA was extracted from the filtered supernatant mentioned in 2.2 using the TRIzol™ Reagent (Invitrogen, USA) according to the instructions provided by the manufacturer. Then the RNA was used to synthesize cDNA with a cDNA Synthesis Kit (Catalog Number: R212-01) purchased from Vazyme (Nanjing, China). The PCR was performed with the 2 × Taq Master Mix (Catalog Number: P111-03) purchased from Vazyme, and the primers utilized for the detection of the PEDV *M* gene. The two primers were designed based on the conserved regions of the PEDV *M* gene. The PCR assay used the following primer sequences: *PEDV-M* forward primer: 5′-TGCGTTCTTGTATGGTGTC-3′ and *PEDV-M* reverse primer: 5′-TAGCAACCTTATAGCCCTC-3′. The PCR was conducted under the subsequent conditions: 5 min at 95 °C, followed by 32 cycles consisting of 30 s at 95 °C, 30 s at 60 °C, and 30 s at 72 °C.

### 2.4 Virus isolation and propagation

Vero cells were seeded in T25 culture flasks and cultured in DMEM supplemented with 10% fetal bovine serum (ExCell Bio, China) and 1% penicillin–streptomycin solution (Yeasen, China) at 37 °C with 5% CO_2_ until a confluent monolayer formed. After removing the culture medium, the cells were washed three times with PBS containing 8 μg/ml trypsin (Gibco, USA). Subsequently, 2 ml of tissue homogenate supernatant supplemented with 1% penicillin–streptomycin solution was added, and the mixture was incubated at 37 °C with 5% CO_2_ for 2 h. The cells were then washed three times with PBS, followed by the addition of 5 ml DMEM containing 8 μg/ml trypsin and 1% penicillin–streptomycin solution for continued culture at 37 °C with 5% CO_2_. During the process, cell status was monitored microscopically. When PEDV-characteristic cytopathic effects (CPE) appeared, the cells and culture medium were subjected to three freeze-thaw cycles. Subsequent viral passages followed the same procedure described above.

### 2.5 Virus purification

Vero cells were seeded into 6-well plates and cultured in a 37 °C, 5% CO_2_ cell culture incubator until a confluent monolayer was formed. The viral samples were serially diluted 10-fold with DMEM containing 8 μg/ml of trypsin in sterile 5 ml microcentrifuge tubes before inoculation onto the confluent cell monolayers. The culture supernatant in the 6-well plates was aspirated, and the cells were washed three times with PBS. The diluted virus solution was gently mixed and added to the wells, followed by incubation at 37 °C in a 5% CO_2_ incubator for 2 h. After incubation, the viral inoculum was aspirated, and the cells were washed three times with PBS. Subsequently, the plaque overlay medium was carefully added to avoid bubble formation. The plaque overlay medium was prepared by mixing 2 × MEM and 1.8% low-melting point agarose at a 1:1 ratio, supplemented with trypsin at a final concentration of 8 μg/ml. Prior to its addition to the cells, the plaque overlay medium was heated to 45 °C and then cooled to 37 °C to maintain a liquid state. After the addition of the plaque overlay medium, the 6-well plates were chilled at 4 °C for 10–15 min to solidify the overlay, then transferred back to the 37 °C, 5% CO_2_ incubator for an additional 2–3 days of culture.

After plaque formation, sterilized pipette tips were used to pick individual viral plaques, which were then subjected to three freeze-thaw cycles. Subsequently, the samples were inoculated into Vero cells for passage according to the method mentioned in **2.4**.

### 2.6 Tissue culture infectious dose 50 (TCID_50_) assay

Vero cells were cultured in 6-well plates until they reached nearly 100% confluence. Following this, the cells were infected with PEDV at a multiplicity of infection (MOI) of 0.01, supplemented with 8 μg/ml of trypsin, for varying durations (12, 16, 20, 24, 28, and 32 h). Subsequently, the cells along with the supernatant were collected and stored at −80 °C. The samples were then subjected to three cycles of freezing and thawing, followed by centrifugation at 12,000 rpm for 10 min at a temperature of 4 °C to obtain the harvested supernatant, which was subsequently used for the TCID_50_ assays according to the following procedures.

Vero cells were seeded in 96-well plates and cultured at 37 °C with 5% CO_2_ until a complete monolayer of cells was formed. Following the establishment of the monolayer, virus samples underwent a 10-fold serial dilution in DMEM supplemented with 8 μg/ml of trypsin. This dilution process prepared the samples for inoculation onto the confluent layer of Vero cells. Each dilution was then introduced into eight wells, with 100 μl of the diluted virus added to each well. The plates were maintained at 37 °C with 5% CO_2_ for an incubation period of 2 days. After the incubation period, the presence of syncytium formation, which serves as a characteristic cytopathic effect (CPE) of PEDV infection, was used to identify PEDV-positive wells. The viral titration was subsequently calculated in terms of the TCID_50_, utilizing the Reed–Muench method (22).

### 2.7 Indirect immunofluorescence assay (IFA)

Vero cells were cultivated in 48-well culture plates until they reached approximately 100% confluence. Subsequently, the cells underwent three rounds of washing with PBS to ensure the complete removal of the tissue culture medium. Following this, the cells were infected with PEDV (MOI = 0.01). The viral infection was carried out at 37 °C in a cell incubator supplied with 5% CO_2_ for varying durations. After that, cells were gently washed with pre-chilled PBS and then fixed with 4% paraformaldehyde (Biosharp, China) at 25 °C for 15 min. After being washed three times with pre-cooling PBS, the fixed cells underwent permeabilization treatment using pre-chilled methanol at −20°C for 15 min. Then the cells were washed three times with pre-cooling PBS and blocked using the blocking buffer (PBS buffer that contained 5% bovine serum albumin) at 37 °C for 1 h. Subsequently, the cells underwent three rounds of washing with pre-chilled PBS to ensure the complete removal of blocking buffer and were incubated with the PEDV S protein monoclonal antibody (established by our laboratory) at 37 °C for 1 h. Afterwards, the cells were washed with PBS for three times to thoroughly clear the primary antibody solutions and incubated with an Alexa Fluor^®^ 488 labeled donkey anti-mouse IgG antibody (Abcam, China) under light-protected conditions at 37 °C for 45 min. Then the cells were washed three times with pre-cooling PBS and incubated with DAPI solutions (MCE, China) to stain the cell nuclei under light-protected conditions at 25 °C for 5 min. After being washed three times with PBS under light-protected conditions, the results of IFA were observed and acquired using a fluorescence microscope.

### 2.8 Genome sequencing and phylogenetic analysis

The RNA genome of PEDV the HB-2024 strain was extracted from the viral culture supernatant using the TRIzol™ Reagent (Invitrogen, USA). Then the RNA was used to synthesize cDNA with a long cDNA Synthesis Kit (Catalog Number: R312-01) purchased from Vazyme. Twenty-eight pairs of primers (Supplementary Table S1) were designed for amplifying the whole genome of the HB-2024 strain by PCR with high fidelity DNA polymerase kit (Catalog Number: P521-d1) purchased from Vazyme. The PCR products were subjected to the agarose gel electrophoresis and then purified by a gel extraction kit (OMEGA, USA). The purified products were sequenced by Sangon Biotech (Wuhan, China). The complete genome was assembled according to the consensus sequence between the adjacent products and then submitted to the GenBank database (https://www.ncbi.nlm.nih.gov/genbank/). A sequence alignment of the PEDV S gene nucleotide sequences between the HB-2024 strain with 95 reference strains was conducted by MEGA 11 software. The neighbor-joining method embedded in MEGA 11 was used to conduct the phylogenetic tree analysis by analyzing the alignment results. The Evolview online software (https://evolgenius.info) was used to draw the final phylogenetic tree.

### 2.9 The structural analysis of S protein

The S protein structures of HB-2024, YN and GD strains were predicted by using alphafold3 server (https://alphafoldserver.com/). The structure comparison and visualization were conducted by using PyMOL software (version 3.0.0).

### 2.10 Animal experiment

Eight 12-day-old piglets in healthy growth condition and confirmed negative for PEDV antigen and antibody were randomly assigned to two separate groups: a HB-2024 infected group and a control group. Each group comprised four piglets and was housed separately in two different piggeries. The pigs were initially housed for 3 days to allow them to acclimate to the environment and then the piglets in HB-2024 infected group were infected with 1 ml of the HB-2024 strain (10^5^ TCID_50_/ml) through oral gavage. The piglets of the control group were administered a mock inoculation of 1 ml of sterile DMEM at the same time, which served as a negative control in relation to the viral challenge. Upon observing diarrhea in the piglets infected with HB-2024, all piglets were euthanized and subsequently underwent necropsy. Parenchymal organs and various sections of the gastrointestinal tract were collected and preserved in 4% paraformaldehyde (Biosharp, China) for histopathological examination and immunohistochemistry (IHC) analysis. The procedures for histopathology and IHC were consistent with the methods described in our previous study (23). The only modification was the substitution of the primary antibody used in IHC with a monoclonal antibody against the PEDV N protein (established by our laboratory).

## 3 Results

### 3.1 Isolation and identification of PEDV

**Figure 1:**
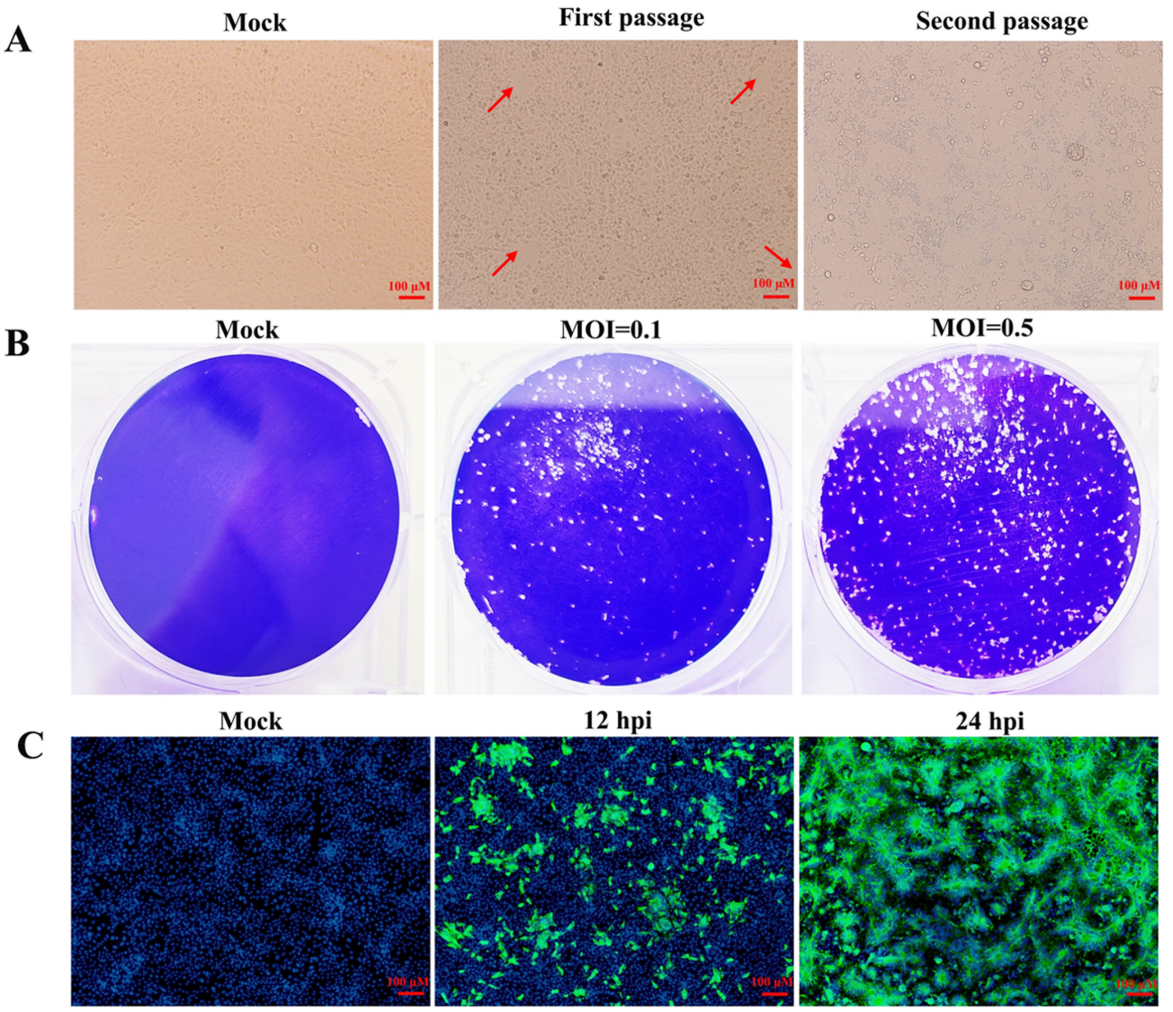
Isolation and identification of the HB-2024 strain. **(A)** Cytopathic effects of the first and second passage in Vero cells. The red arrow indicates the cytopathic effect. **(B)** Plague morphology of the third passage in Vero cells. **(C)** Detection of PEDV S protein by IFA at 12 and 24 hpi in Vero cells.

Before virus isolation, the clinical sample was subjected to RT-PCR to detect the PEDV M gene (Supplementary Figure S1). After confirming the sample as PEDV-positive, it was utilized for isolating PEDV. As illustrated in the Figure 1A, sporadic characteristic lesions emerged during the first passage. In the second passage, after infected with the first passage for 24 h, most of the cells exhibited significant cytopathic effects (CPE), including syncytium formation and cell shedding. Therefore, the plaque formation assay was used to purify the isolated PEDV during the third passage. As shown in Figure 1B, cells infected with the second passage exhibited distinct plaques at 30 hpi. The purified PEDV was achieved by picking individual plaque and inoculating it into Vero cells and was named as HB-2024. Vero cells inoculated with the HB-2024 strain (the second passage after purification) were further examined by IFA. The specific green fluorescence for PEDV S protein and the syncytia were observed (Figure 1C), which demonstrated that a purified PEDV strain was isolated.

### 3.2 Phylogenetic tree of the HB-2024 strain

**Figure 2:**
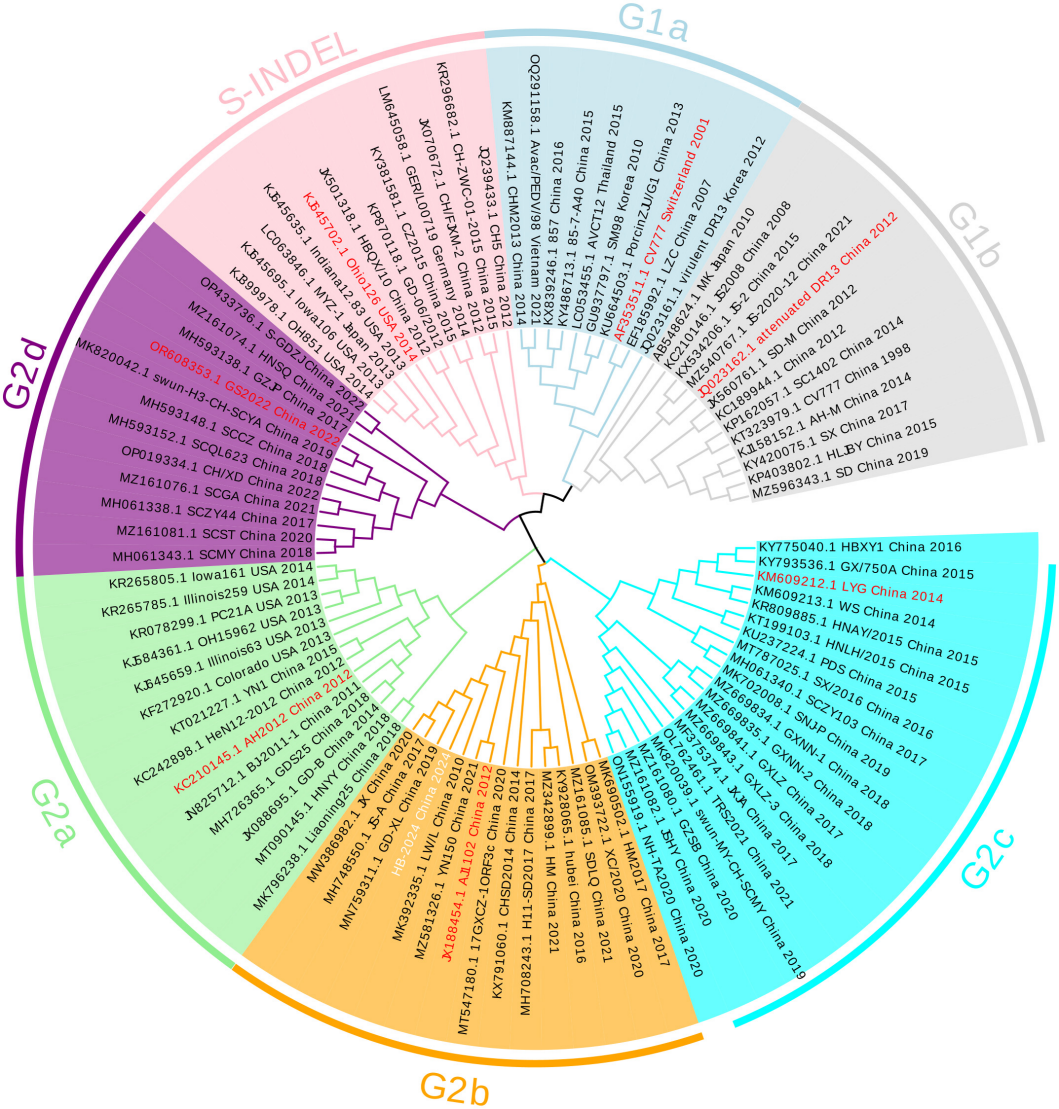
The phylogenetic tree of the HB-2024 strain. The phylogenetic tree was constructed using the neighbor-joining method in MEGA 11 software, incorporating the complete S gene of the HB-2024 strain alongside 95 global reference strains. The final visualization of the tree was generated using Evolview online software. In this visualization, the representative strain of each PEDV subtype is displayed in red font, while the HB-2024 strain is presented in white font.

The whole genome of HB-2024 (the second passage after purification) was identified by assembling the sequencing results of the RT-PCR products for amplifying PEDV genome and uploaded to GenBank with the accession number of PV695983. The S gene of the HB-2024 strain is 4,155 nt and encodes a 1,385-aa protein. The S gene of the HB-2024 strain was used to do a phylogenetic tree analysis with the S gene of 95 reference PEDV strains. As shown in Figure 2, HB-2024 belongs to the G2b subtype.

### 3.3 The growth characterization of the HB-2024 strain in Vero cell

**Figure 3:**
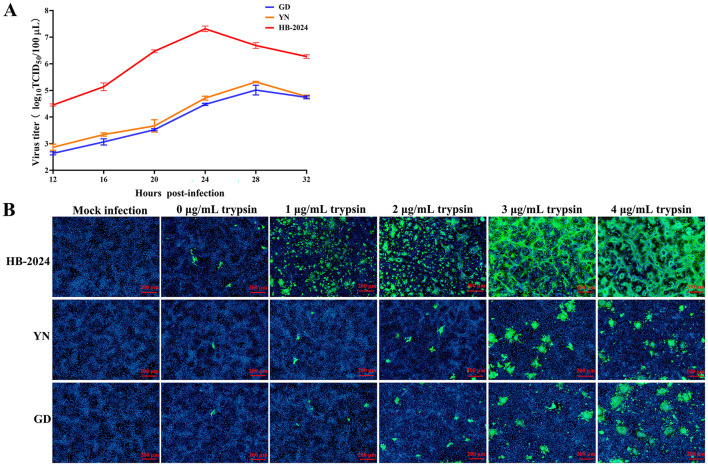
The growth characterization of the HB-2024 strain in Vero cell. **(A)** Growth kinetics of HB-2024, YN and GD strains in Vero cells. Vero cells were infected with HB-2024, YN or GD strain at a MOI of 0.01. Samples were collected at 12, 16, 20, 24, 28 and 32 hpi and used for the TCID_50_ assay. Data are shown as the means ± SD of three independent experiments with the error bars representing the standard deviations. **(B)** Trypsin dependence of HB-2024, YN and GD strains in Vero cells. Vero cells were infected with HB-2024, YN or GD strain at a MOI of 0.01 in the presence of trypsin at the concentration of 0, 1, 2, 3 and 4 μg/ml trypsin. Samples were collected at 24 hpi and used for the IFA.

The growth characterization of the HB-2024 strain in Vero cell was evaluated the one-step growth curve method, with two parallel G2b PEDV strains serving as the experimental control. The one-step growth curve results demonstrated that the viral titer of the HB-2024 strain increased more rapidly, with its peak titer being significantly higher than that of the YN and GD strains (Figure 3A). We also investigated the trypsin dependence of the HB-2024 strain infection by IFA. As shown in Figure 3B, PEDV-S protein-specific green fluorescence could be detected in all PEDV strains infected cells regardless of whether trypsin is added. However, the infection efficiency of the three PEDV strains exhibits significant trypsin dependence. In addition, the HB-2024 strain infection induced more serious and larger CPE than the other two strains at the same trypsin concentration and the same time post-infection, suggesting a low trypsin dependence than the other two strains.

### 3.4 S Protein sequence analysis

**Figure 4:**
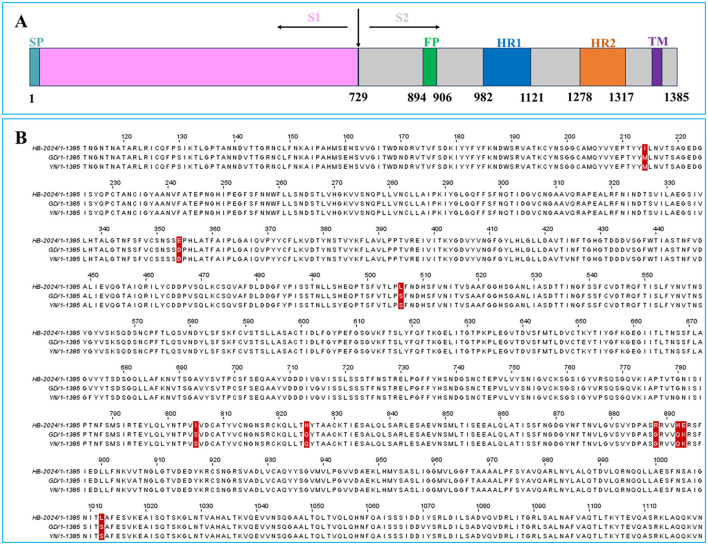
The S Protein sequence analysis of HB-2024, YN and GD strains. **(A)** Schematic representation of the PEDV S protein. SP, signal peptide; FP, fusion peptide; HR1, heptad repeat 1; HR2, heptad repeat 2; TM, transmembrane domain. **(B)** Analysis of amino acid mutations in the S protein of PEDV HB-2024 strain. The amino acid sequences of the S proteins of HB-2024, GD and YN strains were compared using the CLUSTAL W method by MEGA software. The unique mutation sites of the HB-2024 strain are highlighted with a red background.

Our result indicated that the HB-2024 strain possessed high adaptability to Vero cell. Since S protein play vital roles in PEDV adaptability to host cell, the amino acid sequence of HB-2024 was aligned with that of YN and GD strain. Compared to the GD and YN strains, the S protein of the HB-2024 strain harbors nine unique mutation sites: M214I, D354E, S506L, S805I, Q825H, G888R, Q892H, K893E, and S1012L (Figure 4B). According to the functional domains of PEDV S protein (Figure 4A), most mutation sites (6/9) were located in S2 domain. In addition, three adjacent mutation sites (G888R, Q892H, and K893E) were located just beside the N terminal to fusion peptide, which might lead to the higher adaptability of HB-2024 to Vero cells than the other two strains.

### 3.5 Protein structure analysis

**Figure 5:**
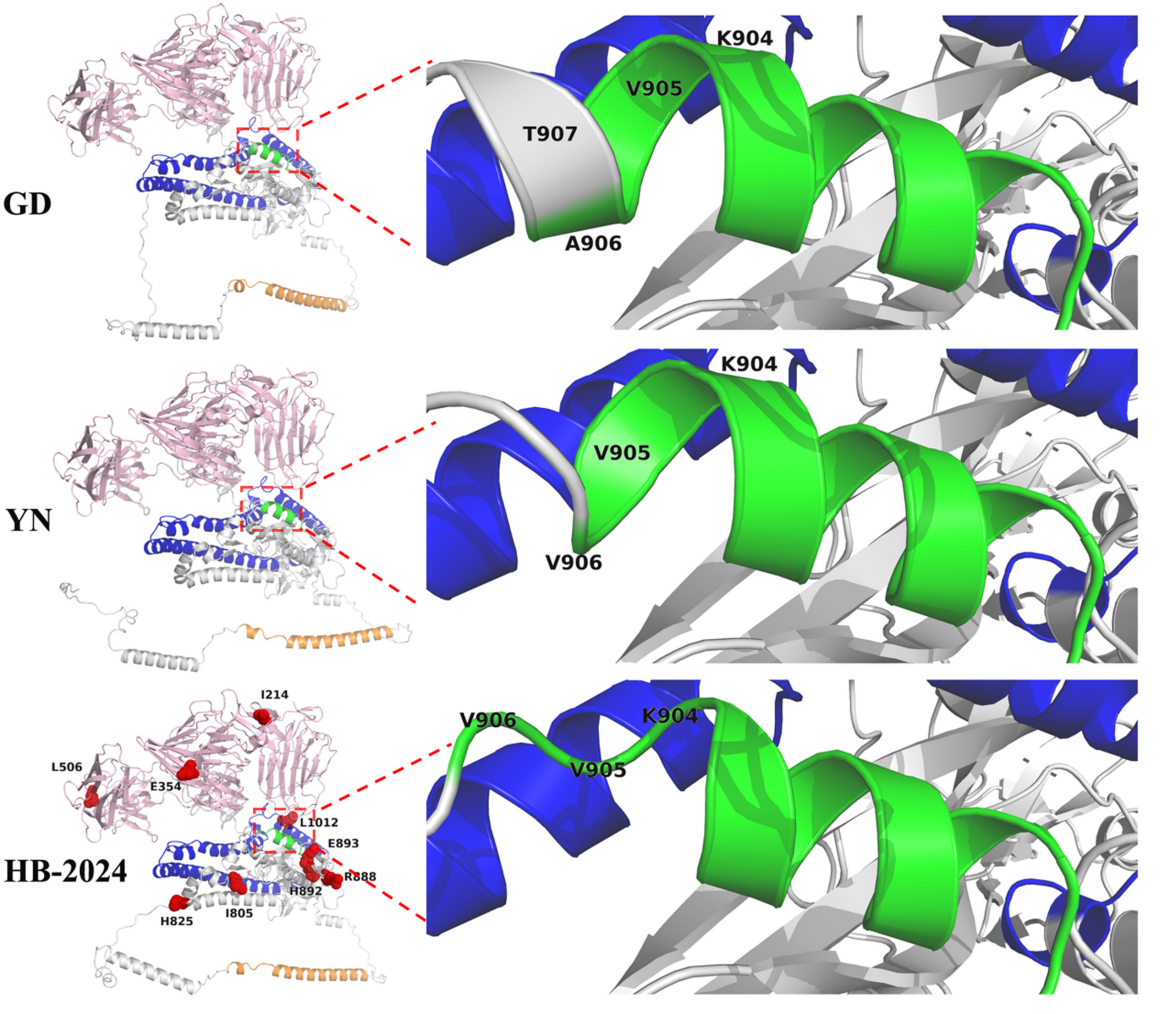
S Protein structure analysis of HB-2024, YN and GD strains. The S1 and S2 domains of S protein are highlighted as pink and gray, while the FP, HR1 and HR2 in S2 domain are shown as green, blue and orange, respectively. The nine unique mutation sites of the HB-2024 strain are shown as red dot in the HB-2024 structural model.

To investigate whether the nine unique amino acid mutation sites of the HB-2024 strain could induce conformational changes in the S protein, AlphaFold 3 was used to predict the 3D structures of the S proteins from PEDV YN, GD and HB-2024 strains. As shown in Figure 5, we found that the alpha helix formed by the FP sequence of the HB-2024 strain's S protein was the shortest among the three S proteins analyzed. Compared to the S proteins of the other two strains, the three amino acids (904RVV906) at the carboxyl terminus of the FP sequence did not participate in forming the alpha helix but instead contributed to the formation of a loop.

### 3.6 Pathogenicity of the HB-2024 strain

**Figure 6:**
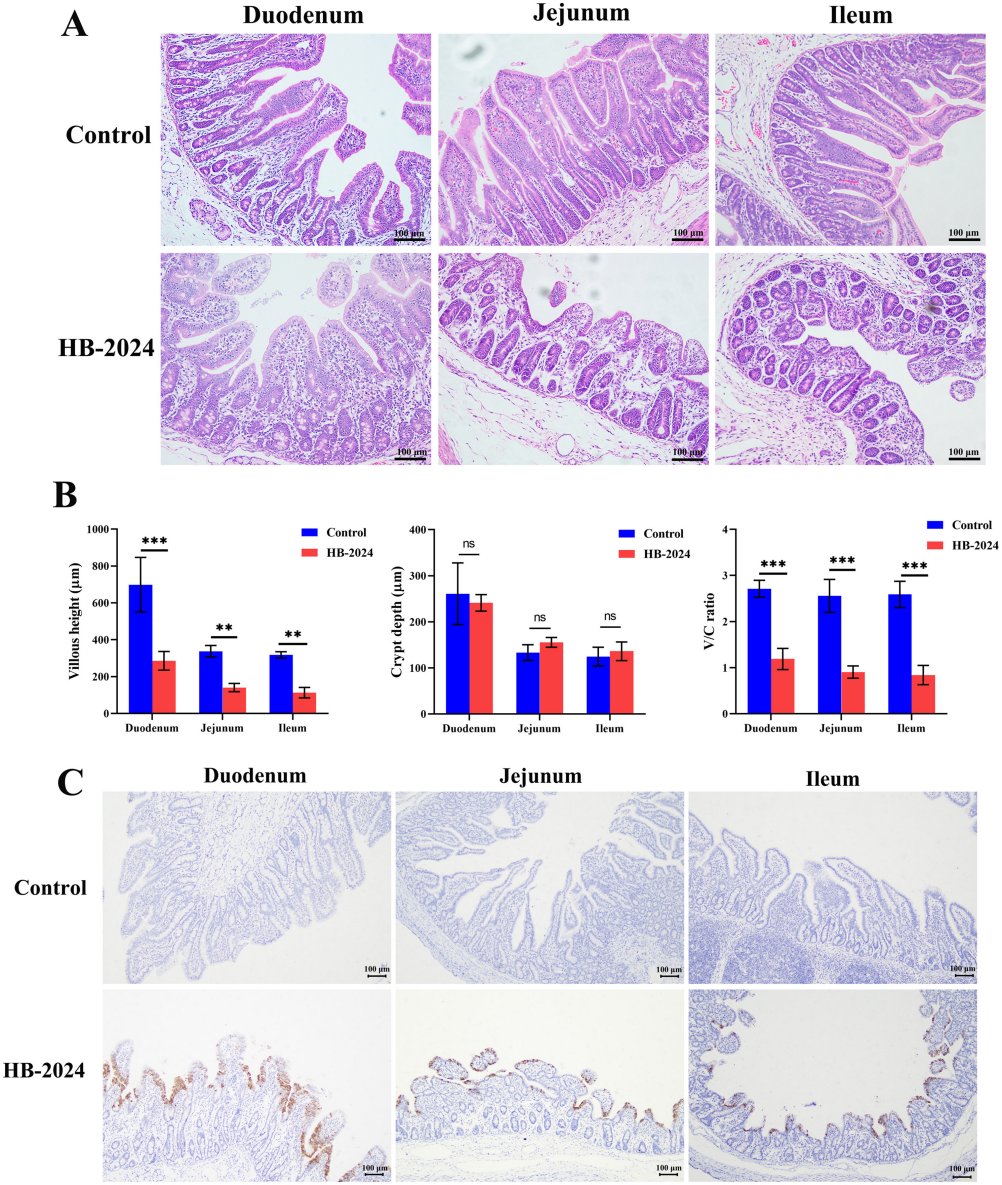
The pathogenesis and tissue distribution of the HB-2024 strain in piglets. **(A)** Histopathological analysis of the HB-2024-infected and control groups. **(B)** The Villous height, crypt depth, and V/C ratio of small intestine in HB-2024 infected and control groups. Data are shown as the means ± SD of four piglets with the error bars representing the standard deviations. Statistical significance was determined using Student's *t*-test (SPSS 17.0) with the following conventions: ^ns^*p* ≥ 0.05, **p < 0.01, and ***p < 0.001. **(C)** The tissue distribution of the HB-2024 strain detected by IHC.

All piglets infected with the HB-2024 strain showed severe watery diarrhea by 2 days after infection, whereas those in the control group did not exhibit any clinical symptoms. The autopsy results revealed that piglets infected with HB-2024 presented with gastric distension, thinning and transparency of the intestinal walls, along with intestinal lumens filled with undigested milk clots (Supplementary Figure S2). Pathological alterations were observed in the duodenum, jejunum, and ileum of the PEDV-infected group, which included irregularities and shedding of epithelial cells, defects and irregularities in the striated border, as well as the coarsening and shortening of villi (Figure 6A). The villous height, crypt depth, and villous height and crypt depth (V/C) ratio were also measured to demonstrate the intestinal lesions caused by HB-2024 infection. As illustrated in Figure 6B, the HB-2024 infected group displayed a significant reduction in villous height and the V/C ratio in the duodenum, jejunum, and ileum compared to the control group, accompanied by an increase in crypt depth. IHC for detecting PEDV N protein was used to explore the tissue distribution of the HB-2024 strain. Results from IHC indicated the presence of PEDV in the intestinal epithelial cells of the small intestine (Figure 6C).

## 4 Discussion

PEDV is currently one of the most significant diarrheal pathogens threatening the global swine industry. Due to its single-stranded positive-sense RNA genome, it exhibits considerable variability. Since its initial discovery in 1971, PEDV has evolved into three major genotypes: G1, G2, and S-INDEL, with the G2 genotype was the high-pathogenic has been the dominant epidemic strain since 2010.

In this study, we isolated a PEDV strain from the intestinal tissues of clinically diarrheic piglets. Phylogenetic analysis revealed that this strain belongs to the G2b genotype. Notably, during the isolation process, we observed distinct differences in its growth characteristics compared to our previously isolated strains in Vero cells. Sporadic Vero cells exhibited typical pathological changes specific to PEDV infection during the first passage, whereas most of the cells demonstrated such changes at 24 hpi in the second passage. Compared to two previously isolated PEDV G2b strains, the HB-2024 strain exhibited earlier and more severe CPE at the same MOI and the same time post-infection. One-step growth curve analysis revealed that the HB-2024 strain achieved peak viral titers in a shorter time frame, with peak titers more than 2 log_10_ TCID_50_/100 μl higher than those of the other two strains. Furthermore, compared to the other two strains, the HB-2024 strain exhibited lower dependency on trypsin for infecting Vero cells. All these findings indicate that the HB-2024 strain possesses significantly stronger adaptability to Vero cells than the other two strains.

S protein is closely associated with the cellular adaptability of coronaviruses (24, 25). As a highly glycosylated protein, the S protein exists as a homotrimer on the outermost layer of coronaviruses (26). During coronavirus invasion, the S protein mediates viral attachment to host cells and initiates membrane fusion (27). The membrane fusion process is induced by the S2 subunit, though the intact S protein cannot directly trigger fusion (28, 29). Following viral receptor binding, host proteases cleave the S protein to expose the S2 subunit, thereby activating membrane fusion. The PEDV S protein can also be cleaved by a variety ominf proteases to facilitate entry, especially by trypsin, which plays a critical role in PEDV isolation (30–32). The cleavage site of trypsin on PEDV S protein is located near the fusion peptide, where variations in specific amino acids may influence trypsin-mediated S protein cleavage efficiency (7, 33, 34) and consequently affect viral cell adaptability. In this study, the S protein of the HB-2024 strain exhibited nine unique amino acid mutations compared to two other strains. Among these, three adjacent mutations clustered near the fusion peptide region, suggesting their potential role in enhancing the adaptability of HB-2024 in Vero cell. Furthermore, Tan et al. demonstrated that trypsin preferentially cleaves at arginine residues proximal to the fusion peptide, and the presence of arginine at these sites facilitates S protein cleavage, thereby promoting membrane fusion and syncytium formation—the characteristic CPE of PEDV infection (34). Notably, the HB-2024 strain showed a G-to-R substitution at position 888 of the S protein compared to the other two strains, further supporting that amino acid divergence in the fusion peptide region underlies its superior cell adaptability.

It is well-known that after multiple *in vitro* passages, viruses usually exhibit significantly enhanced cell adaptability but reduced pathogenicity in natural host. The HB-2024 strain demonstrates strong cell adaptability, yet whether it induces disease in piglets remains to be investigated. Animal experiments demonstrated that all 15-day-old piglets challenged with the HB-2024 strain developed severe diarrhea, and pathological results revealed significant lesions in the small intestine of infected pigs. Consistent with the previous studies (23, 35), immunohistochemical results indicated that the HB-2024 strain predominantly localized in the intestinal villus epithelial cells of the duodenum, jejunum, and ileum. These findings confirm that the HB-2024 strain retains certain pathogenicity. However, due to the absence of corresponding control strains, the correlation between the cell adaptability of the HB-2024 strain and its pathogenicity remains undetermined. Subsequent studies will initially utilize reverse genetics technology to construct recombinant viruses and identify the key domain or amino acid in S protein responsible for the high cell adaptability of the HB-2024 strain. Following this, animal experiments will be conducted to elucidate the relationship between the adaptability of this strain and its virulence.

In conclusion, we isolated a highly cell-adapted PEDV G2b strain and evaluated its pathogenicity through animal experiments. Amino acid sequence analysis revealed that the unique signatures near the fusion peptide region of the HB-2024 strain may serve as the key determinants of its high cell adaptability. These findings provide valuable insights for further elucidating the mechanisms underlying PEDV cellular adaptation and guiding PEDV vaccine development.

## Data Availability

The original contributions presented in the study are included in the article/Supplementary material, further inquiries can be directed to the corresponding authors.
