# Supplementary material for: Isolation and pathogenicity of a variant porcine epidemic diarrhea virus field strain with high adaptability to Vero cell
Source: Front Vet Sci. 2025 Aug 26;12:1654230. doi: 10.3389/fvets.2025.1654230 (PMC12417201; doi:10.3389/fvets.2025.1654230)
Supplement: Supplementary file 1 [file Table_1.docx]

Table S1. Primers for amplifying PEDV whole genome

| **Primer name** | **Primer sequence (5’-3’)** |
| --- | --- |
| 1F | ACTTAAAGAGATTTTCTATCTATG |
| 1R | CACCTTAAGAATTCGCCAGAATGC |
| 2F | ATTGGACTTCCTATGTCTCCAC |
| 2R | ACACAAAGCTCTGCAACACTAG |
| 3F | TCTTCGAGAGTGACGGGTTTTAC |
| 3R | CCTCAATGGCAACGTTGATATACTC |
| 4F | GTGGTGGTGATGTCAAATTCTCTG |
| 4R | CAAGGTCACTAAAATCTCCTTGG |
| 5F | GGTTATGGTCGTCATCAGATAAAG |
| 5R | GTAACAGTGGATTCTACTTTAACAG |
| 6F | CAAGGTATCTAAGCTTAAAGGCA |
| 6R | GGTACACCAGCTAGAACTTTAAC |
| 7F | CAGACGGCTGTTGTGATTAAAGAC |
| 7R | GCATTCTTAACCTGATTTACATTGG |
| 8F | GTCCTGCCACTATTCTTATTGAC |
| 8R | CAAGCCGAATTAAAAATGCAAGC |
| 9F | CTATAGTCGTTGGTGTATCAGAC |
| 9R | AGCCAGCCTGTAGAGTTGAATTG |
| 10F | CTGCGTCAGTATGCTAGTACTT |
| 10R | CAGGATTTACCCAAAAGAACTTAG |
| 11F | CTATACCTCGTTGACAGATGAG |
| 11R | GGCGTGCATTTTCATAAATGACA |
| 12F | TGTGTAATGACCCAGAAAAAGC |
| 12R | CATTACCAGAACCATTGGCCAAC |
| 13F | ACATAGGTGCCACTGTACGCT |
| 13R | GGTATACCCATTCCCTTGATGCT |
| 14F | CCTACTTCAATAATAAAGTCTGGT |
| 14R | TGATGATACTGCCGAGTAGTCA |
| 15F | TCCTTTGAACAAGTTTGGTAAAG |
| 15R | CAGAATTTAGCAGTAGAATCTTCC |
| 16F | GCTTGAACGTTATGTGTCATTGG |
| 16R | CAAAGTGAATCGACCGCTGCA |
| 17F | CATACCTGAAGCTTATTCTAGC |
| 17R | CGAGATAACATGCTCATATTTAATG |
| 18F | GCTTAAATTGTCTGATTTGCAGG |
| 18R | CACATTACAATTCCAAAACAAGC |
| 19F | CTTAAGTTATACAATCCGAAAGC |
| 19R | ATGTCGCTACGAGGGCTAAAATC |
| 20F | ACTGGCATAAAGTTAACTTATGG |
| 20R | GTACAGAACATTGTCCAATACTC |
| 21F | CCTTATACCTGTCAGATAAGTTTG |
| 21R | GTTCGTAAACATATTGCATAGCAC |
| 22F | CTATTTAACAAAGCCATCCCAGC |
| 22R | GTGAAATGGTAAATTGTCTAGTGTC |
| 23F | CCAACTCAAGTGTTCTCAGGT |
| 23R | GAAACACCCAGCACATTAGTA |
| 24F | TACACTGCAGCATGTAAGACC |
| 24R | AACGGTAGGTTTTCTAGGTTC |
| 25F | CATTTTCTCTCTGGTACAGGC |
| 25R | CCCACGTATAGCTAGATACAAG |
| 26F | TGGCGCTATAAAAATGCGCTC |
| 26R | GATTACTCACAGCTGAGTAGTCG |
| 27F | CACTCCTTAGTGGTACATTGC |
| 27R | GCATCTCCAAAATTTTTGAAGCC |
| 28F | GCCCTTAAATCTTTGGGTATTG |
| 28R | GTGTATCCATATCAACACCGTCA |
