# Supplementary material for: Isolation and pathogenicity of a variant porcine epidemic diarrhea virus field strain with high adaptability to Vero cell
Source: Front Vet Sci. 2025 Aug 26;12:1654230. doi: 10.3389/fvets.2025.1654230 (PMC12417201; doi:10.3389/fvets.2025.1654230)
Supplement: Supplementary file 2 [file Image_1.pdf]

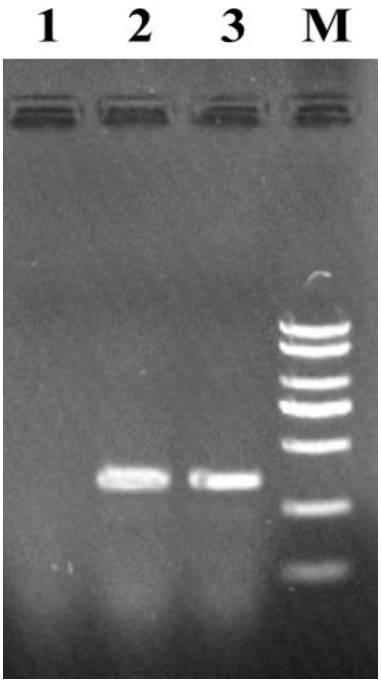

Figure S1. Identification of PEDV in clinical samples by RT-PCR. 1, negative control; 2, positive control; 3, the clinical sample; M, DL2000.

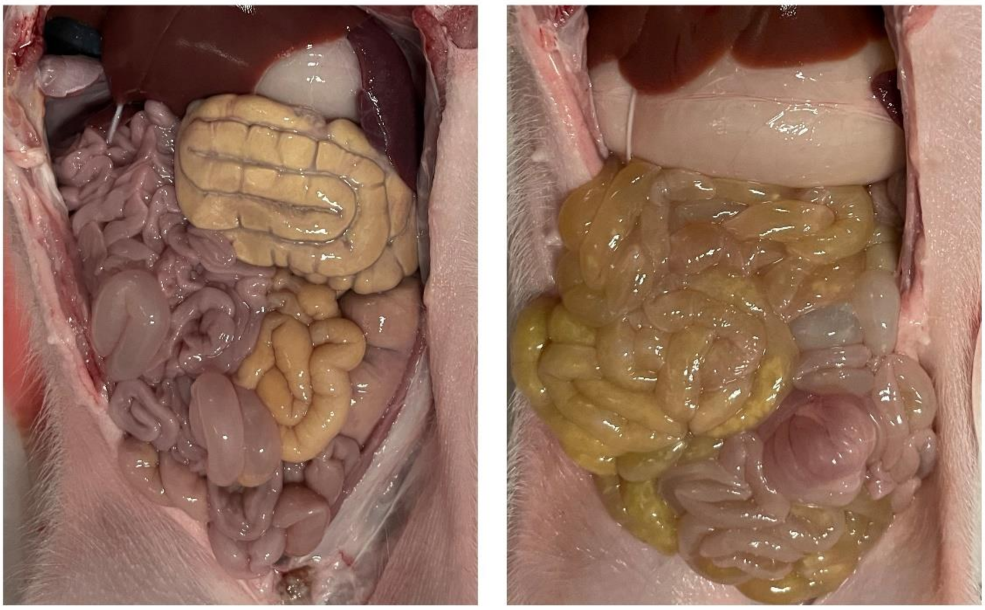

Figure S2. The gross anatomy and organ lesions of piglets in the control group and HB-2024 infected group.
